# Pulp Mummification

**Published:** 1900-01-15

**Authors:** Owen E. Houghton

**Affiliations:** Brooklyn, N. Y.


### PULP MUMMIFICATION.

BY OWEN E. HOUGHTON, D.D.S., BROOKLYN, N. Y.

Read before the Second District Dental Society, October, 1899.

About twenty-five years ago, in a fit of desperation over difficulties met in trying to remove the devitalized pulp from the roots of an upper third molar or wisdom tooth, I concluded to leave the pulp in the roots and watch results.

The pulp-chamber was carefully wiped with carbolic acid, and then filled with os-artificial (oxychloride of zinc), and the usual amalgam filling finished the operation.

No inflammation or tenderness followed, and that tooth remained a member in good standing in the dental society of its fellows for many years after.

Another wisdom tooth was soon after treated in the same way, with equally good results.

Gradually, I came to treat all wisdom teeth in this manner, when the pulp needed to be destroyed. I found that the general result was that the percentage of failure, or after trouble, was less in these teeth than in teeth where I had given my best skill and patience in trying to fill all root canals to their very ends. It was exceedingly rare I ever had trouble with a wisdom tooth so treated.

I never applied the term pulp mummification to this treatment, but called it pulp preservation.

I had not the courage, except in isolated cases, to extend the treatment to other teeth, and continued for years to inflict the tortures of the nerve broach and drill upon my patients, in a conscientious effort to practice what was supposed to be the best methods taught by my dental college, my dental society and the text-books, often with the result of exhausting the patience and nerves of both myself and patient, only to realize after all, in spite of my best skill and efforts, I had not and could not fill every root perfectly or to my satisfaction. Pericemental inflammation has, I believe, often been started from "monkeying" too much with the ends of root canals, especially in freshly devitalized pulps.

For years I have read with great interest all that has been printed in our dental journals about pulp preservation or pulp mummification.

The cobalt method of Herbst, of which Dr. Bodecker, wrote such a glowing account, made a great impression on me, but I could not see wherein it was superior to the oxychloride of zinc method I had practiced for so many years with good results, and cobalt being a first cousin to arsenic itself, I did not dare to trust it.

When Dr. Soderborg, of Sidney, Australia, published his formula for a pulp mummifying paste, giving his method of treatment, the results of several experiments, and his experience for one year in its successful application, it was, to my mind, the ideal formula I had so long looked for.

I began the use of this paste for pulp mummification in July, 1896. Up to date, I have the diagram record of three hundred and seventeen teeth where the pulps had been devitalized by me, and this paste used for mummification of pulps left in root canals. This number does not include shedding teeth so treated by me, as I keep no record of operations on these teeth.

Of the three hundred and seventeen cases on record, I have yet to report the first failure, not a single abscess or discolored tooth, except from the amalgam filling, not one lost, to my knowledge. There is scarcely a day in my general practice I do not see one or more of these teeth, and have always found them in perfect health.

These treatments were not confined to molars and bicuspids, but cuspids and incisors also were treated by this method. They were not confined to any class of patients; rich and poor, old and young, sick and well, were subjected to the same treatment.

I have had but two opportunities to open any of these teeth after treatment.

First case was a left upper second molar treated six months before. In attempting to extract the wisdom tooth root adjoining this tooth, my forceps broke out the filling. I removed the oxyphosphate and paste, and found the root canals vacant as far as I could discover. The paste was replaced and tooth filled as before. This was two years ago. That tooth is all right to-day.

Second case was where filling broke out of lower first molar. Again the canals empty and no odor except a perceptible one of thymol. This tooth had been treated two years before.

My mode of treatment differs but little from that practiced by Drs. Soderborg and Waas. I will give it, as I think some useful details, have been omitted by both.

After the pulp is devitalized, I open up the chamber, remove its dead contents, and all I can of the pulp in the opening of the canals that can be reached and removed by the ordinary wire explorer. Always keep chamber sterilized with dental meditrina, then dry out thoroughly with hot air syringe, fill entire pulp-chamber with mummifying paste and carefully seal with oxyphosphate cement. Fill the tooth with whatever material you prefer, and dismiss your patient with the assurance that the tooth will never bother again.

In bicuspid teeth, I frequently enlarge the pulp-chamber with a bur that I may be able to put in a larger quantity of the mummifying paste. With incisors and cuspid teeth, I bur out pulp half way up the canal, desiccate, fill empty part of the canal with the paste, seal up with oxyphosphate and fill with gold.

You may wonder why I treat the single canal teeth in this manner, when the pulps can be easily removed and the canals filled.

I will be candid, gentlemen. I positively believe from my experience that I run much less danger of pericemental inflammation when I leave a small portion of the pulp in the end of the root and mummify it, than I do to tear it away with a nerve broach, or guess at its length with a drill (we thought it dead, but our patient knew better); and again the operation of filling the canal of the root is seldom *perfectly* accomplished, and even when it is so done, you have, on some occasions, been rewarded with an abscess.

It was my fortune to treat the left superior lateral tooth for a lady a few weeks ago. I found a small alveolar abscess at end of root. Patient stated the root had been filled twenty years ago, and by one of the best operators in this State. The abscess had been there from the first year the tooth had been filled. I opened up the canal and removed as perfect a gold root filling as I imagine can be made by the most expert. I mention this only to prove that our most careful and perfect root fillings do not always prevent abscess.

It is possible that, in no distant future, some of my mummified pulps may be heard from in form of an abscess. If it does occur, I can assure you I will have no trouble in getting into the canal and treating the tooth.

To those of you that have but little faith in pulp mummification, let me recommend that you try it on shedding molars of children, where the pulp becomes exposed and the little one comes to you with an aching tooth. Destroy and remove pulp from chamber only, fill chamber with mummifying paste, and fill cavity with oxyphosphate. You will find this a most successful method of treating such teeth.

I have my mummifying paste made up fresh about every two months. In about that time it becomes so dry and stiff as to be hardly workable. At the end of six months, it is hard and dry as gunpowder.

As the devitalization of the dental pulp is so important a factor in this mode of treatment, and so much has been written about the evils attending the application of arsenic to the dental pulp, I will here give you the formula that has been used by myself, and my uncle before me, for more than forty years.

| | | |
|---|---|---|
| ℞. | Acetate of morphia . . . | 20 grs. |
| | Arsenious acid . . . . . | 10 grs. |
| | Tannin . . . . . . . . | 10 grs. |

Triturate thoroughly.

Apply to exposed pulp on small pellet of cotton saturated with carbolic acid, seal with oxyphosphate or soft gutta-percha.

This preparation has been uniformly successful in my hands. I would recommend its renewal every year at least, as in time the arsenic is liable, owing to its great specific gravity, to become separated from the other ingredients.

In pulp mummification it is seldom necessary to make a second application of the arsenic. The pulp in the chamber can, as a rule, be burred out without pain, while that in the canals still retain considerable vitality. I do not hesitate to introduce the paste in the chamber and seal up a tooth in this condition, rather than make the second application of arsenic. Am careful not to use much pressure. The only evil result experienced in such a case is a slight feeling of uneasiness for a day or two. That 's the last you hear of it.

I have given you my experience in pulp mummification and admit it is a most radical departure from the regular method of dental practice, but I feel assured it will never lead up to such disappointment as copper amalgam and cataphoresis brought to hasty advocates.

Dr. Waas used the same method as myself, and began his treatment at the same time, i. e., January, 1896. He gave his experience in the treatment of sixty-one teeth with not one failure to record. He had then had two and one-half years' experience. In this paper I supplement his testimony with three hundred and seventeen cases, and nearly four years' experience in pulp mummification.

Most of you will remember, and it was not long ago, how the wise men of our profession taught that it was "*a crime to destroy the dental pulp.*" Most of us are to-day reaping a harvest of putrescent pulps, pericementitis and alveolar abscess from attempting to live up to such teachings. Capped pulps were the principal causes of these disasters. The death of the pulp after the tooth has once been filled is, to-day, I believe, the cause of more suffering and loss of our patients, and more anxiety and loss of reputation to ourselves, than all other causes put together in the dental line. In the future I do not intend to take the chances I have in the past, in capping pulps.

The following interesting comments were made on Dr. Houghton's paper at the meeting when read:

DR. D. W. BARKER: If the thymol paste is made in a quantity of say four drams, as the formula requires, a large proportion is wasted because it soon hardens, and though it may be softened with glycerine, it is a crumbly mass and unfit for use. A better way is to obtain the ingredients (thymol, glycerine, dried alum and oxide of zinc) separately and mix a small quantity as needed. Second, do not try to push the paste into a canal by cotton-shod broaches or any other instrument. The easiest and also the most thorough way is to place in the pulp-chamber a pellet of the paste, then rubb it into the canals with a pellet of cotton held in the pliers. If one will take an extracted tooth, clean out the canals and try the experiment of rubbing the paste into the canals with cotton pellets, he will be surprised how quickly the paste will be carried through the canals.

DR. A. H. BROCKWAY: I have made use of this method quite generally for more than three years, and with increasing confidence. I do not recall a single case where it has proved unsatisfactory either to myself or my patient. That the work involved is much less tedious to both goes without saying, since usually not more than two short visits, at the most, are required to accomplish equally good, if not better, results than were formerly secured by repeated and tedious sittings when it was deemed essential to remove every shred of pulp tissue from even the most minute root canals, and afterward to fill them with various solid substances from gold to gutta-percha.

Should the case be one involving a dead and putrescent pulp, there is, in my experience, no treatment so simple and efficacious as that described by Dr. Schreier with the use of kalium natrium at first, followed, if necessary, by cleaning, disinfecting and such treatment as may be needed to put the root in a thoroughly aseptic condition.

Dr. W. J. Turner: One hundred percent of successes is perfection, and that is what we are all striving for. However, the future may show that some of these apparent successes may fall short of that ideal, and abscesses may develop. We know this happens after our most careful and approved methods of root filling, and then our peace of mind is proportional to the ease of removal of the particular kind of root filling employed. Dr. Houghton mentions having had several opportunities of examining the condition of the root canals after his method of treatment, and in each instance has found them empty, exactly as we would wish them to be for the easy treatment of alveolar abscess. I believe there are but few who deal with many cases of pulpless teeth who can show such an unbroken line of successes with them, and when we add to this the very favorable condition of the root canal for further treatment, should this ever be necessary, it would seem that this is the best method yet given to us.

Dr. W. Andre Campbell: Dr. Herbst, by his method, also claimed a perfect immunity from future trouble, and possibly it is not owing so much to the properties contained in Dr. Houghton's mummifying paste that makes it appear so successful as the method employed for devitalizing the pulp, as both used arsenic, and both filled the tooth before a septic condition could possibly interfere.

Dr. E. T. Rippier: I have treated several cases after the manner described in the paper. After devitalizing the pulp I removed it from the chamber which was there enlarged and the mummifying paste applied and covered with a disc of asbestos paper upon which the filling is inserted. All the cases I have treated are so far successful.

Dr. F. P. Hamlet: The mummification of the pulp, or the mummification of matter remaining in a tooth, after the removal of the pulp, is not an impossibility, as we have learned by Dr. Houghton's report of over three hundred cases treated by him.

My experience in the use of this paste covers almost three years—using it (not exclusively) with some precaution. The devitalized pulp should be removed as soon as possible, if allowed to remain long the functions of the tooth will become diseased by the presence of the decomposing pulp.

We can in no better way complete this symposium than by reprinting Dr. Kirk's editorial in *The Dental Cosmos* for May, 1899, as it suggests the wisdom of careful experimentation before we accept a course so radically opposite to that which is known to be conservative.

